# The development and validation of a decision aid to facilitate patient choice of surgery versus radiotherapy for high‐risk basal cell carcinoma

**DOI:** 10.1111/ced.15325

**Published:** 2022-08-12

**Authors:** Jamie Banks, Joy Odili, Shane Zaidi, Susan Lalondrelle, Masha Singh, Victoria Akhras, Zainab Jiyad

**Affiliations:** ^1^ Department of Dermatology St George's Hospital London UK; ^2^ Department of Plastic Surgery St George's Hospital London UK; ^3^ St George's University of London Medical School London UK; ^4^ Department of Clinical Oncology The Royal Marsden Hospital Sutton UK; ^5^ Population Health Research Institute St George's University of London London UK

## Abstract

Basal cell carcinoma (BCC) is an increasingly common cancer. For high‐risk BCCs, there are several treatment options, with similar efficacies. The current best practice in deciding upon a particular treatment is for a patient‐centred approach. At present, there are few resources available for patients to assist their choice. This reduces patient autonomy and increases the burden on clinicians within clinic. Patient decision aids (PDAs) have been shown to increase patient autonomy and facilitate shared decision‐making. Currently, there is no published PDA designed to facilitate the decision between surgical management or radiotherapy in high‐risk BCCs. We developed a novel decision aid designed along the International Patient Decision Aid Standards to fill this clinical need, and evaluated its acceptance by both patients and clinicians. We describe the challenges faced at initial alpha and subsequent beta testing, and go on to validate our PDA with both the Decisional Conflict Scale and the nine‐item Shared Decision Making Questionnaire (SDMQ9). We include an example of the PDA and encourage other units to modify the PDA for their own use.

## Graphical abstract

Patient decision aids (PDAs) are increasingly used by clinicians in the setting of shared decision‐making. We present a novel PDA for patients presenting with high‐risk basal cell carcinoma, to aid in their decision between radiotherapy and surgery. We explore the design process for the decision aid and its validation using validated outcome measures, the nine‐item Shared Decision Making Questionnaire and the Decisional Conflict Scale, including an illustration of the example decision aid.

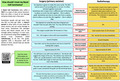

High‐risk basal cell carcinoma (BCC) as defined by recent British Association of Dermatologists (BAD) guidelines, is based on various features such as site of tumour, clinical border, histological subtype and level of invasion.^1^ Surgical options, namely primary excision or Mohs micrographic surgery (MMS), are preferentially recommended in the guidelines, but in practice, radiotherapy (RT) is a frequent treatment option offered to patients aged ≥ 60 years. As suggested by BAD guidelines, RT may be given if the patient expresses a preference for RT over surgery.^1^ Although the 2021 guidelines note increased recurrence rates (with one trial noting a 10‐fold increase in recurrence rates^2^), they also note the acceptance of RT as a treatment option across a number of international guidelines.^1^ Indeed, a 2018 systematic review suggested that the recurrence rate with radiotherapy of 3.5% was comparable to the standard of primary excision or MMS, both of which have a recurrence rate of 3.8%.^3^ A range of specialists may be involved in treatment discussions, including plastic surgeons and oncologists, and patients are often given a sizeable amount of information at a single consultation, including the diagnosis and treatment options, as well as potential risks.

As shared decision‐making becomes the gold standard for clinicians, patient decision aids (PDAs) have been adopted across the clinical spectrum. These have been shown to increase knowledge of a condition and its treatment, and encourage patients to consider the advantages and disadvantages of the various treatment options.^4^ The most recently updated Cochrane Review found evidence indicating positive effects when decision aids were used, either within or in preparation for the consultation.^4^

## Report

We identified a clinical need for improved information dissemination for patients diagnosed with BCC and facing the decision between surgery (primary excision) and RT. Thus, during the period May–August 2021, we developed a novel PDA following the principles set out by the International Patient Decision Aid Standards.^5^ A literature search was conducted to provide accurate evidence‐based data for the PDA.

Alpha testing was undertaken with feedback from clinicians and patients. Subsequently, we validated this decision aid in beta testing, using two validated outcome measures, the nine‐item Shared Decision Making Questionnaire (SDMQ9) and the Decisional Conflict Scale (DCS).^6^, ^7^ We also assessed the overall benefit of the PDA using a patient satisfaction questionnaire (PSQ) to compare experiences pre‐ and post‐implementation, based on negative framing of questions (Supplementary Fig. S1). Responses were graded on a Likert scale, and examples of questions included were: ‘I feel I was overwhelmed with the information I was given today’; ‘I don't feel I have a clear understanding of the risks and benefits of each treatment’; and ‘I felt rushed to make a decision today’.

Average response scores were calculated with 95% CI, and following the Jarque–Bera test to confirm normality, Student *t*‐test was used to compare pre‐ and post‐decision aid scores.

After an initial draft of the PDA was created, alpha testing prompted several modifications. Including the addition of two questions (‘How many hospital visits are needed?’ and ‘Where is it done?’), as well as a QR link to the British Association of Dermatologists patient information leaflet on BCC. Complexity of language was also discussed. The average reading age of the UK public has been estimated at < 12 years.^8^ Accordingly, we adapted the text to simplify its language, and we estimated a final readability age between 8 and 12 years old, based on widely‐available readability calculators: Flesch Reading Ease Score, Gunning Fog Index, Flesch–Kinkaid Grade Level, Automated Readability Index and Coleman–Liau Index.

**Figure 1:**
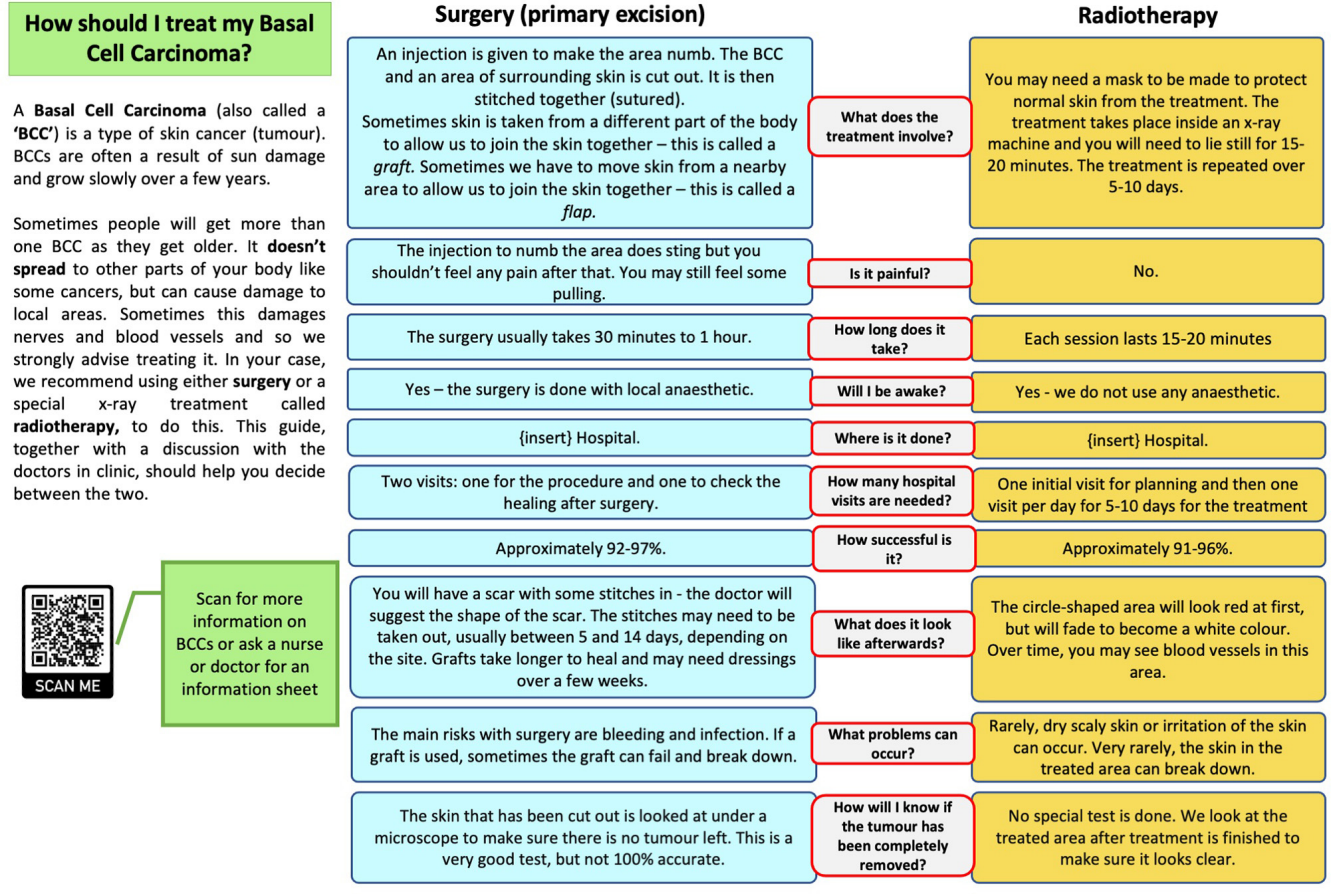
Decision aid, primary excision vs. radiotherapy for high‐risk basal cell carcinoma (BCC). [Colour figure can be viewed at wileyonlinelibrary.com]

The final version of the PDA (Fig. 1; available for free distribution and modification) was then developed and distributed at clinic attendance to 18 consecutive patients who were referred to a combined clinic for consideration of surgery vs. RT for BCC, together with the PSQ and example photographs to demonstrate post‐surgery and post‐RT outcomes. We also distributed the PSQ to 18 consecutive patients who were similarly referred to the combined clinic prior to PDA development. As per BAD guidelines, patients aged < 60 years, patients deemed eligible for MMS, and patients with recurrent BCCs or BCCs on limbs, were not offered RT.^1^

Post‐implementation, we found the mean DCS response across the beta testing cohort of 14 complete responses was 1.70 out of 5 (95% CI 1.60–1.79), i.e. a score between ‘strongly agree’ and ‘agree’ that the PDA aided in deciding on a treatment. We received 17 responses for the SDMQ9, with mean of 4.93 out of 5 (95% CI 4.71–5.15), i.e. between ‘somewhat agree’ and ‘strongly agree’ (tending towards the latter) that the PDA facilitated shared decision‐making. The PSQ was completed by 17 patients who received the PDA, with mean response of 1.62 out of 5 (95% CI 1.51–1.74), i.e. a score between ‘disagree’ and ‘strongly disagree’ that there was excessive/inadequate information or that they felt rushed into a decision. This represented a modest but significant (*P* < 0.001) mean improvement of 0.75 points vs. pre‐PDA implementation, which had a mean value of 2.38 out of 5 (95% CI 2.20–2.56).

A large volume of information is given to patients diagnosed with BCC, including prognosis, treatment options and potential benefits/risks of each treatment option. A PDA given in advance of a multidisciplinary combined clinic, usually when giving the histopathology result, allows the patient time to review the options and discuss with family and/or friends. This decision aid has been developed and validated to facilitate the choice of primary excision vs. RT. We encourage its use in outpatient settings if both options are available and equitable. In general, we encourage use of PDAs to enhance patient‐centred care and facilitate shared decision‐making.

## Conflict of interest

The authors declare that they have no conflicts of interest.

## Funding

None.

## Ethics statement

Ethics approval and informed consent not applicable.

## Supporting information


**Supplementary Figure S1** Patient satisfaction questionnaire.

## Data Availability

Data are available on request from the corresponding author.
