# Supplementary figures and images for: The development and validation of a decision aid to facilitate patient choice of surgery versus radiotherapy for high‐risk basal cell carcinoma
Source: Clin Exp Dermatol. 2022 Aug 12;47(11):1995–7. doi: 10.1111/ced.15325 (PMC9804260; doi:10.1111/ced.15325)

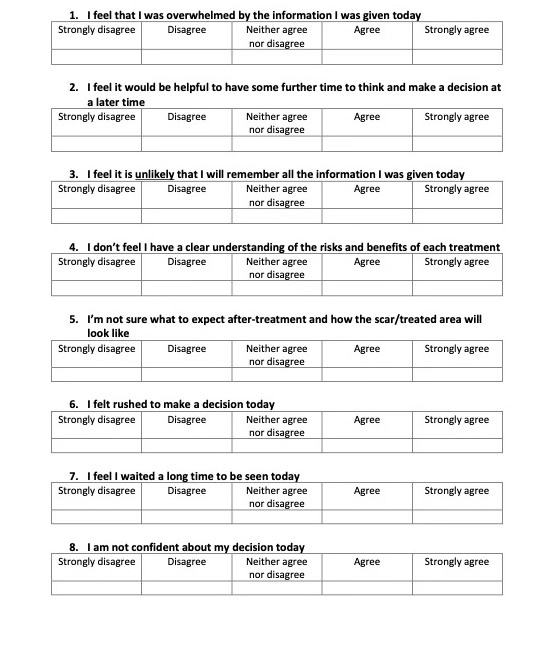

Supplement: Supplementary file 1 — Supplementary Figure S1 Patient satisfaction questionnaire. [file CED-47-1995-s001.jpeg]
